# Breastfeeding is associated with reduction in postpartum depression in the United Arab Emirates: a retrospective cross-sectional study

**DOI:** 10.1038/s41598-025-94912-3

**Published:** 2025-03-27

**Authors:** Ala Al Rajabi, Hind Alkatheeri, Rafiq Hijazi, Lynne Kennedy

**Affiliations:** 1 Department of Nutrition Sciences, College of Health Science, QU Health, Qatar University, Doha, Qatar; 2 Department of Health Sciences, College of Natural and Health Sciences, Zayed University, Abu Dhabi, UAE; 3 Department of Mathematics and Statistics, College of Natural and Health Sciences, Zayed University, Abu Dhabi, UAE; 4 Department of Public Health, College of Health Science, QU Health, Qatar University, Doha, Qatar

**Keywords:** Breastfeeding, Postpartum depression, EPDS, Physical activity, United Arab Emirates, Human behaviour, Risk factors, Depression, Lifestyle modification

## Abstract

Postpartum Depression (PPD) is a common mental health disorder affecting mothers. Breastfeeding may be protective against PPD. Global estimates of breastfeeding and PPD rates vary, especially for women living in Middle Eastern countries. The current study aims to assess breastfeeding and PPD prevalence and to identify factors associated with reduced PPD risk within the social and cultural contexts of the UAE. We used a purposive, convenience snowball sampling technique to recruit participants. Inclusion criteria were female ≥ 18 years, mother of a child ≤ three years, and resident of Abu Dhabi, UAE. Data was collected using an online survey distributed via email and social media platforms. The survey comprised four sections: sociodemographic characteristics, breastfeeding behaviour, Edinburgh Postnatal Depression Scale (EPDS), and The International Physical Activity Questionnaire –Short Form (IPAQ-SF). Pearson chi-squared tests and binary logistic regression model were used to investigate the associations between PPD levels and potential predictors using SPSS statistical software. Variables included in the regression model were breastfeeding duration, delivery mode, BMI, education, general health, physical activity level, employment status, number of children, and age. All statistical significance was considered at p-value < 0.05. In total 403 subjects consented to participate; 204 met the inclusion criteria and were included in the final analysis (age [mean ± SD] = 31.2 ± 7.3 years). Among them, 34.8% suffered from moderate-to-severe PPD, and 66.2% breastfed their last child for > 3 months. Regression model results showed that (OR; 95% CI) college education (0.39; 0.19–0.80), having more than one child (0.40; 0.17–0.94), self-reported very good (0.43; 0.19–0.98) and excellent health (0.21; 0.08–0.51), and breastfeeding for > three months (0.46; 0.23–0.92), were significantly associated with reduced odds of moderate-to-severe PPD. None of the remaining variables -including physical activity- were significant. In conclusion, breastfeeding is significantly associated with a reduction in moderate-to-severe PPD among mothers in Abu Dhabi, UAE.

## Introduction

Mental health disorders, including depression, are among the leading causes of ill-health and premature death globally, including in the United Arab Emirates (UAE)^1,2^. Worldwide, approximately 970 million people experience mental ill-health^3^, with one in three women experiencing mental ill-health during their lifetime^4^. Postpartum Depression (PPD) is a common mental health disorder affecting many women, resulting in mild to severe health issues for mother and child. Research indicates that breastfeeding may be a protective factor in preventing PPD and should therefore be encouraged to promote future health and well-being of all new mothers. The protective benefits of breastfeeding are however equivocal, as the effect is both variable and bi-directional. The problem is confounded by issues of measurement for breastfeeding duration and PPD diagnosis; precise estimates of breastfeeding rates and PPD prevalence are therefore lacking, with many studies suggesting considerable variations exist between women, including studies conducted in the same region. We also know that PPD prevalence and breastfeeding rates are socially and culturally situated; geography, ethnicity, socio-cultural factors, number of children, age, and self-reported general health are associated with the variations reported. Thus, a better understanding of the association between PPD and breastfeeding will require studies that are socially and culturally situated. Only a limited number of studies have been conducted in the UAE; moreover, as a country of rapid economic development including social and cultural transition, a better understanding of the association between breastfeeding and PPD among women living in the UAE is important, to inform future messaging on breastfeeding and mental health.

## Background

**Postpartum depression (PPD)** is a debilitating Major Depressive Disorder (MDD), which arises in the 4-weeks following childbirth and could last up to three years, with symptoms ranging from negligible to severe^5,6^. PPD is characterized by decreased interest in daily life and routine activities including self-care and lack of interest in the baby. Therefore; mothers who experience PPD typically struggle to care for their newborn resulting in attachment issues that may affect subsequent cognitive and emotional well-being of children^5^. Furthermore, women who suffer from PPD are more likely to experience more serious mental health conditions in the future^6^.

### PPD etiology

The etiology of PPD is well-documented and the risk factors are widely acknowledged^7^. Key factors include, but are not limited to, a history of psychological disorder, pre-existing history of depression and anxiety including during pregnancy, a lack of social support, stressful life events, relationships with significant others, low economic status, complicated delivery, baby’s birthweight, marital conflict, and women’s employment status. Furthermore, a study conducted in Dubai, UAE,^8^ indicates the importance of stressful life events, baby’s birthweight, marital conflict, and women’s employment status as predictors of moderate to severe PPD.

### PPD prevalence

PPD diagnosis involves clinical classification, yet the signs and symptoms are largely symptomatic sharing similarities with generalizable health concerns. One example is how the majority of new mothers experience the so-called ‘baby blues’, resulting from considerable physiological and hormonal changes during pregnancy, childbirth, and the immediate postnatal period^6^. Not surprisingly, PPD is commonly misclassified by healthcare professionals and new mothers. This may also account for the wide-ranging PPD prevalence reported within the global literature. A review based on 143 studies estimated global PPD prevalence to range between 0.5 and 60%^9^. Others suggest a modest (17.7%) PPD prevalence for women in Western countries^10^; studies involving women living in Northern European and Scandinavian countries suggest an even lower prevalence, at 10%^11^. A recent comprehensive review involving over 500 studies published in Nature^12^ confirms wide-ranging global estimates of PPD prevalence, with women in high-income countries experiencing lower rates (15%), compared with women in low- and middle-income countries (15.3–25.7%). In comparison, studies suggest higher PPD prevalence amongst women living in Arab and Middle Eastern countries. A meta-analysis of 15 studies reported a pooled PPD prevalence estimate of 27% for Middle Eastern countries^7^. This resonates with two UAE studies, which suggest a higher PPD prevalence amongst women in this region, ranging between 31 and 44%^13^, and subsequent reports of 33%^8^. Both articles cite social and cultural context as key determining factors. In Islam, women are encouraged to breastfeed for 2 years and various passages in the Holy Quran (Surah Al-Baqarah 2:233) and Hadith state the importance of this practice. As such, UAE culture strongly promotes breastfeeding.^13^ A greater understanding of protective behaviours, and the role played by social and cultural factors, is therefore important.

### Breastfeeding and PPD

The health benefits of breastfeeding up to the age of 6 months, or as long as possible, for mother and child, are also widely accepted. There is a bi-directional association between breastfeeding and PPD; research suggests that breastfeeding is associated with lower PPD rates and higher PPD levels are associated with no breastfeeding or shorter breastfeeding duration^14–18^. A study conducted in the Middle East found that mothers who breastfed were less likely to develop PPD compared with mothers who did not breastfeed^16^. In another study, breastfeeding was linked to significantly better mood and lower levels of perceived stress^19^. Several studies have found an association between the duration of breastfeeding and lower risk of PPD^14,20^ Women who breastfeed their babies also report fewer cases of moderate to severe PPD^21^. The World Health Organization (WHO) recommends women should exclusively breastfeed their child for a minimum of 6 months, and partial thereafter for the greatest health benefits^22^. Whilst we know that breastfeeding duration is important, the literature also suggests that any breastfeeding, regardless of exclusivity, may offer protection against PPD and is therefore worth examining.

### Breastfeeding prevalence in UAE and other countries

For most countries, breastfeeding initiation rates are moderate to high, falling dramatically with time; while exclusive breastfeeding rates fall sharply. In a US study, 85% of mothers initiated breastfeeding but only 48% continued to 3 months^23^. A German study reported 90% of mothers initiated breastfeeding, but this fell to 61% after 4 months^24^. Reportedly one in five (20.5%) women in the Middle East exclusively breastfed^25^; whilst another study conducted in the UAE also reported high initiation rates (70.3%), only 1:4 (26.7%) women were breastfeeding exclusively at 6 months^26^. Whilst further consideration is given to factors associated with breastfeeding in the discussion, we know that socio-cultural factors such as age, occupation, educational attainment, and factors such as number of children and vaginal or caesarian birth are all associated with breastfeeding duration and exclusivity, and as such were included in the current research study. The goal of the current study is to assess factors associated with PPD, including breastfeeding duration, among mothers in the UAE.

## Methods

### Study design and population

A cross-sectional study -with retrospective data collection- was conducted to assess the association between breastfeeding behaviour and PPD levels among mothers in Abu Dhabi, UAE. To enhance cultural relevance, only Abu Dhabi residents were included, excluding respondents from other Emirates or outside the UAE. This criterion aimed to ensure a more homogenous sample regarding social and cultural characteristics. Inclusion criteria were: (1) female aged 18 and above, (2) mother of a child three years or younger, (3) Abu Dhabi resident (4) holds active Google account, and (5) has provided consent to participate before answering the survey. A total of 403 responses were received, with 204 mothers included in the analysis after applying the inclusion and exclusion criteria.

### Data collection instrument

Data collection took place during October 2022 utilizing an anonymous online survey (Google Form). A purposive, convenience snowball sampling technique was employed to recruit participants who met the inclusion criteria. Invitations were sent via the university-wide email list to all female students at Zayed University. Recipients were encouraged to share the survey link with eligible family, friends, and social networks, consistent with snowballing technique. The survey link was also distributed through social media platforms to enhance recruitment.

The survey, created in Google Forms, was embedded in the email invitation and was accessible only after participants completed online informed consent and initial screening. Additionally, to ensure the integrity of the dataset, we applied restrictions in Google Forms to (i) verify participant eligibility (Supplementary Table S1), (ii) require login with a personal Google account to confirm active user status, and (iii) enable the 'Limit to 1 response’ option on Google Forms to prevent duplicate submissions by tracking email addresses. Participants were informed they could exit the study by quitting the survey at any time, without consequence. At the end of the study period, the survey was disabled. The online survey consisted of four sections: sociodemographic characteristics, breastfeeding behaviour, Edinburgh Postnatal Depression Scale (EPDS), and The International Physical Activity Questionnaire – Short Form (IPAQ-SF). All survey questions were provided in Arabic and English, the two main languages used in the UAE (Supplementary Table S1).

### Edinburgh Postnatal Depression Scale (EPDS)

Participants were assessed retrospectively for PPD during the first six weeks following last delivery, using EPDS^27^. All participants included in the study had a child three years or younger. EPDS is a validated questionnaire utilized in clinics and research studies to identify PPD levels^8,28,29^. EPDS is composed of a self-rating 10-item questionnaire with a 4-point Likert scale. Responses range from 0 to 3 (0 = No, not at all; 1 = Not very often; 2 = Yes, quite often; 3 = Yes, most of the time). The total score ranges from 0 to 30: none or minimal depression (0–6), mild depression (7–13), moderate depression (14–19), and severe depression (19–30)^30^. The current study used English and validated Arabic versions of EPDS from Alberta Health Service^31^. The phrasing was slightly modified to fit the study timeframe and its retrospective nature. The Cronbach’s alpha of the EPDS scale was found to be 0.8939.

### The International Physical Activity Questionnaire—Short Form (IPAQ-SF)

Self-reported physical activity during the first 3–6 weeks after the last delivery was assessed using the validated IPAQ-SF^32,33^. We collected information on the frequency and duration of time spent weekly walking and engaging in moderate and vigorous physical activities across recreational, household, transport, and occupational domains. Weekly minutes of walking and moderate- and vigorous-intensity activities were calculated independently. Calculated minutes/week in each category were multiplied by the corresponding metabolic equivalent of task (MET; multiples of resting energy expenditure. Walking = 3.3 MET, moderate intensity = 4.0 MET, vigorous intensity = 8.0 MET), then summed resulting in a total physical activity estimate expressed as MET minutes of physical activity/week (MET- minutes/week)^32^. Participants were categorized into low physical activity (< 600 total MET- minutes/week) vs. moderate-to-high physical activity (≥ 600 total MET- minutes/week)^34^.

### Sociodemographic, clinical, and breastfeeding data

We collected data on participants’ current age, weight, height, marital status, level of education, occupation, self-rated general health, and number of children. We also collected last delivery-specific data including mode of delivery, sex of the last child, breastfeeding duration, and reasons for not breastfeeding or stopping breastfeeding before six months as recommended by the WHO.^35^.

### Data analysis

The sociodemographic characteristics of participants are presented as mean (standard deviation, SD) for age, and count (percent) for categorical variables. Pearson chi-squared test or Fisher’s exact test of independence was used to test the association between PPD levels (none-mild vs. moderate-severe) with different study variables. All statistical significance was considered at p-value < 0.05.

A power analysis was conducted using G*Power for binary logistic regression to assess the adequacy of our sample size (n = 204). The analysis was based on the observed odds ratio for breastfeeding duration (OR = 0.46), the probability of having Moderate to Severe depression (0.348), and the variance explained by other predictors (R^2^ = 0.14). Using a significance level of 0.05, the computed statistical power was 0.995, indicating that our study had the sufficient power to detect meaningful size effects. We ran a binary logistic regression model to further investigate the associations between PPD levels and potential predictors, with PPD level (none-mild vs. moderate-severe) as the dependent variable. None-mild PPD level was assigned as the reference level for PPD level analysis. The estimated associations are presented as odds ratios (ORs) and 95% confidence intervals (CIs). In the regression model, we adjusted for breastfeeding duration (≤ three months; > three months), delivery mode (vaginal delivery; cesarean delivery), BMI (underweight/normal weight; overweight/obese), the highest level of education (college degree; no college degree), general health (good/ fair; very good; excellent), physical activity level (low physical activity; moderate-to-high physical activity), employment status (employed; unemployed), number of children (one; > one ), age in years (20–29; ≥ 30). The criterion for statistical significance was set at alpha < 0.05 (2-tailed). All analyses were performed by SPSS statistical software (SPSS 28.0).

### Ethical approval

Ethical clearance was obtained from Zayed University Research Ethics Committee (Reference number: ZU23_017_F). The study was conducted in accordance with Helsinki Declaration guidelines^36^.

## Results

### Sociodemographic characteristics

A total of 204 women residing in Abu Dhabi, UAE met the inclusion criteria and were included in the final analyses (age [mean ± SD] = 31.2 ± 7.3 years old). As shown in Table 1, the majority of the participants were Emiratis (84.3%), Middle Eastern (94.6%), aged between 20 and 39 (84.3%), married and/or living with a partner (94.6%), well-educated (58.3%), unemployed (74.5%), overweight or obese (57.4%), reported moderate-to-high physical activity levels (52.0%) and reported ‘very good’ or ‘excellent’ general health (76.0%).

**Table 1:** Sociodemographic, clinical, and breastfeeding characteristics of the study population (n = 204 women).

Variable	Categories	Count (percentage)
Nationality	Emirati	172 (84.3%)
	Other nationalities	32 (15.7%)
Ethnicity	Middle Eastern	193 (94.6%)
	Far East Asian	5 (2.5%)
	Southeast Asian	1 (0.5%)
	African	3 (1.5%)
	Caucasian/White	2 (1%)
Age (years)	20–29	95 (46.6%)
	30–39	77 (37.7%)
	40 +	32 (15.7%)
BMI (Kg/m^2^)	Underweight (< 18.5)	9 (4.4%)
	Normal weight (≥ 18.5 and < 25.0)	78 (38.2%)
	Overweight (≥ 25.0 and < 30.0)	74 (36.3%)
	Obese (≥ 30.0)	43 (21.1%)
Marital status	Married and/or living with a partner	193 (94.6%)
	Single^$^	11 (5.4%)
Education	Less than high school	10 (4.9%)
	High School	75 (36.8%)
	Associate degree/diploma	26 (12.7%)
	Bachelor’s degree or higher	93 (45.6%)
Employment status	Employed	52 (25.5%)
	Unemployed	152 (74.5%)
General Health	Good /fair	49 (24%)
	Very good	83 (40.7%)
	Excellent	72 (35.3%)
Number of children	One	61 (29.9%)
	More than one	143 (70.1%)
Physical activity^£^	Low	98 (48.0%)
	Moderate-to-high	106 (52.0%)
Delivery mode^¥^	Vaginal delivery	162 (79.4%)
	Cesarean section	42 (20.6%)
Child’s sex^¥^	Male	114 (55.9%)
	Female	90 (44.1%)
Breastfeeding duration^¥^	≤ 3 months^€^	69 (33.8%)
	> 3 months	135 (66.2%)

^$^ Single includes: divorced, separated, and single/never married.

^£^ Participants were categorized into low physical activity (< 600 total MET- minutes/week) vs. moderate-to-high physical activity (≥ 600 total MET- minutes/week).

^¥^ Related to the last delivery.

^€^ Never breastfed or breastfed for 3 months or less.

### Clinical and breastfeeding data

Regarding the last delivery, most of the participants delivered their babies via vaginal delivery (79.4%), had a male child (55.9%), and breastfed their infant for more than three months (66.2%), as presented in Table 1.

### Reasons for not breastfeeding for six months or more

**Fig. 1:**
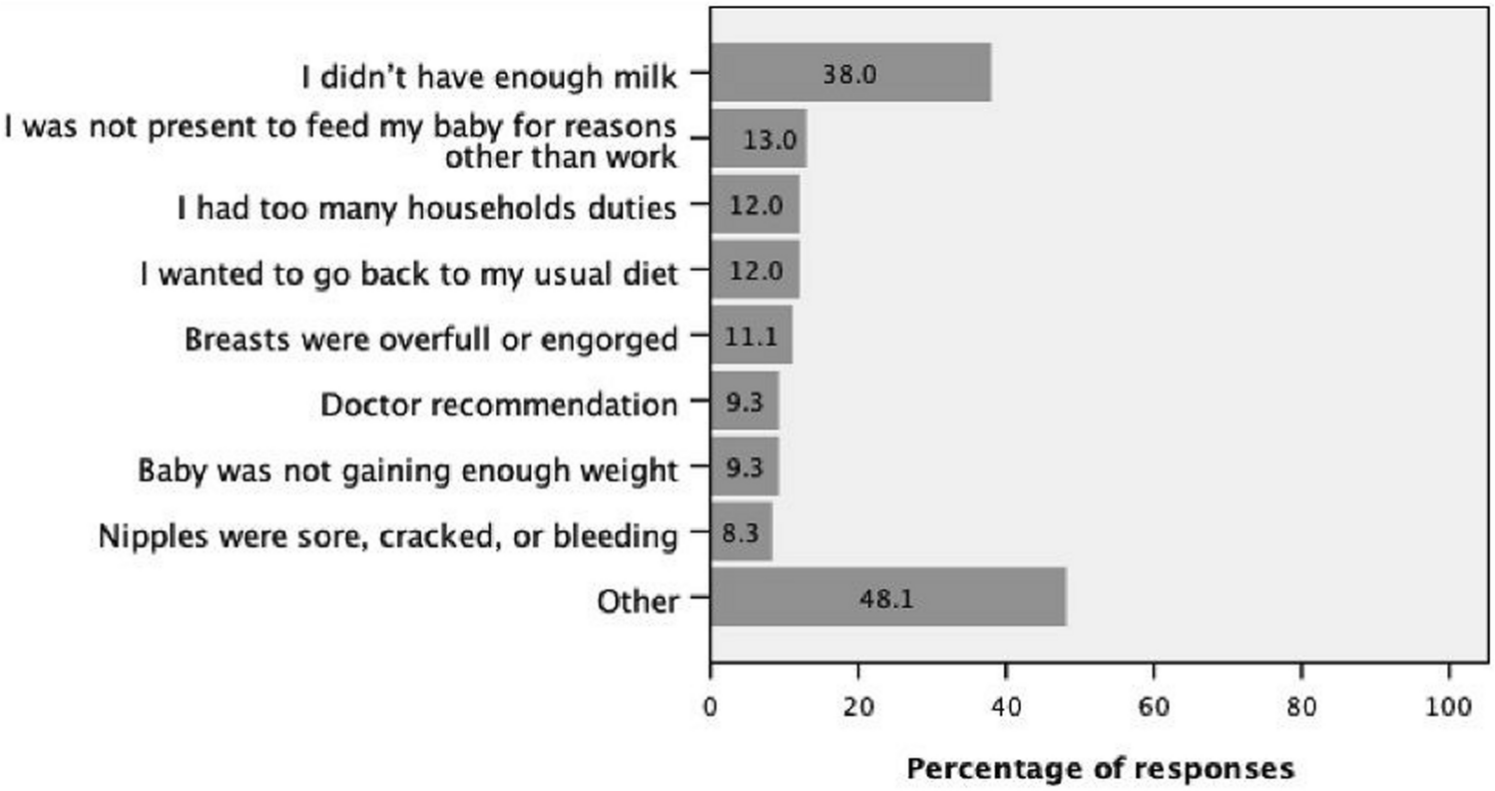
Reasons for not breastfeeding or stopping breastfeeding before six months. Mothers who did not breastfeed their infants or stopped breastfeeding before six months of age (n = 73) were asked to select all applicable reasons from a provided list.

We asked mothers who did not breastfeed their infants or stopped breastfeeding before six months of age (n = 73) to choose the reason (select all that apply). As is shown in Fig. 1, the main reason was “I didn’t have enough milk”, which was selected by 38.0% of participants. Other reasons were selected by less than 15% of participants including: “I wanted to go back to my regular diet” (12.0%), “I had too many household duties” (12.0%), “baby was not gaining enough weight” (9.3%), and “Doctor recommendation” (9.3%).

### EPDS responses and participants’ score

**Fig. 2:**
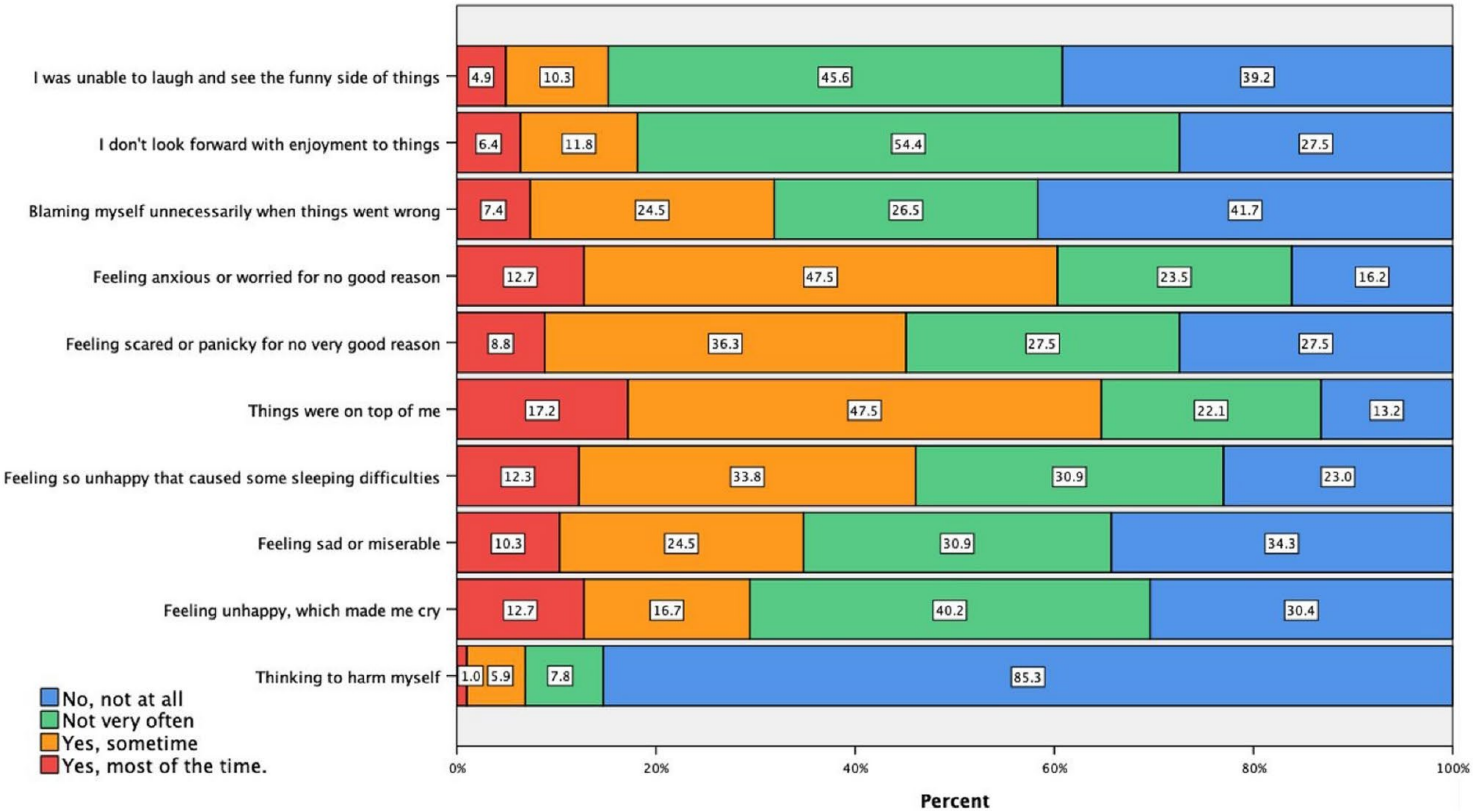
EPDS responses and participants’ score. All study participants (n = 204 women) completed the EPDS questionnaire, with responses indicating the frequency of experiencing various postpartum depression symptoms.

Figure 2 shows participants’ responses to EPDS individual items. A considerable proportion of participants (≥ 30%) responded “yes, sometimes” or “yes, most of the time” to 7 out of the 10 EPDS items, indicating experiencing the particular EPDS item. Among participants, 64.7% and 60.2% responded “yes, sometimes” or “yes, most of the time” to “things were on top of me” and “feeling anxious or worried for no good reason”, respectively. On the other hand, only 6.9% of participants gave the same responses to “thinking to harm myself”, indicating that self-harm crossed their minds.

EPDS total score ranges from 0 to 30: none or minimal depression (0–6), mild depression (7–13), moderate depression (14–19), and severe depression (19–30). Overall, total EPDS score was (mean ± SD) 11.08 ± 6.44. For the subsequent analysis, the EPDS score was categorized as none-mild depression (0–13) and moderate-severe depression (14–30). Overall, 133 (65.2%) participants scored none-mild PPD (7.3 ± 4.0), while 77 (34.8%) participants suffered from moderate-severe PPD (18.1 ± 3.6).

### Prevalence and associations of PPD across study variables

In Table 2, we present prevalence of PPD levels (none-mild vs. moderate-severe) and its associations across different study variables. The significance of associations was tested using Pearson chi-squared test or Fisher’s exact test.

**Table 2:** Prevalence of postpartum depression levels (none-mild vs. moderate-severe) and its association with different study variables (n = 204; Chi-squared).

Variable	Categories	Level of depression [N (%)]		Chi-squared P-value
		None-Mild	Moderate- Severe	
Nationality	Emirati	113 (65.7%)	59 (34.3%)	0.727
	Other nationalities	20 (62.5%)	12 (37.5%)	
Ethnicity	Middle Eastern	127 (65.8%)	66 (34.2%)	0.520^a^
	Others	6 (54.5%)	5 (45.5%)	
Age (years)	20—29	51 (53.7%)	44 (46.3%)	0.001*
	30 +	82 (75.2%)	27 (24.8%)	
BMI (Kg/m^2^)	Overweight/Obese (≥ 25.0)	80 (69.6%)	35 (30.4%)	0.136
	Underweight/Normal weight (< 25.0)	53 (59.6%)	36 (40.4%)	
Marital status	Married and/or living with a partner	128 (66.3%)	65 (33.7%)	0.196^a^
	Single^$^	5 (45.5%)	6 (54.5%)	
Education	No college degree	68 (61.3%)	43 (38.7%)	0.197
	College degree	65 (69.9%)	28 (30.1%)	
Employment status	Employed	33 (63.5%)	19 (36.5%)	0.761
	Unemployed	100 (65.7%)	52 (34.3%)	
General health	Good /fair	23 (46.9%)	26 (53.1%)	0.002*
	Very good	54 (65%)	29 (35%)	
	Excellent	56 (77.7%)	16 (22.3%)	
Number of children	One	30 (49.2%)	31 (50.8%)	0.002*
	More than one	103 (72.0%)	40 (28.0%)	
Physical activity^£^	Low	67 (68.4%)	31 (31.6%)	0.361
	Moderate to High	66 (62.2%)	40 (37.8%)	
Delivery mode^¥^	Vaginal delivery	103 (63.5%)	59 (36.5%)	0.341
	Cesarean section	30 (71.5%)	12 (28.5%)	
Child’s sex^¥^	Male	80 (70.2%)	34 (29.8%)	0.093
	Female	53 (58.9%)	37 (41.1%)	
Breastfeeding duration^**¥**^	≤ 3 months^€^	35 (50.7%)	34 (49.3)	0.002*
	> 3 months	98 (72.6%)	37 (27.4%)	

* Statistically significant at *p-value* < 0.05.

^$^ Single includes: divorced, separated, and single/never married.

^£^ Participants were categorized into low physical activity (< 600 total MET- minutes/week) vs. moderate-to-high physical activity (≥ 600 total MET- minutes/week).

^¥^ Related to the last delivery.

^€^ Never breastfed or breastfed for 3 months or less.

^a^ Fisher’s exact test.

Breastfeeding for > 3 months, compared to breastfeeding for ≤ 3 months (27.4% vs. 49.3%; p-value = 0.002), having more than one child, compared to having one child (28.0% vs. 50.8%; p-value = 0.002), older age (p-value = 0.001), and better reported general health (p-value = 0.002), were all significantly associated with reduction in moderate-severe PPD prevalence among study participants. None of the other variables -including employment status, physical activity, delivery mode, BMI, and child sex- reached statistical significance (p-value ≥ 0.05).

### Binary logistic regression model

To further clarify the associations of breastfeeding duration with PPD, we ran a binary logistic regression model with PPD level (none-mild; moderate-severe) as the dependent variable. Table 3 provides odds ratios (ORs) and 95% confidence intervals (CIs) for the variables included in the model, using “none-mild” depression as the reference group. In terms of the performance of this model, Nagelkerke’s R-squared indicated that the model explained 15.8% of the variance in the outcome. The likelihood ratio test confirmed that the model provided a significantly better fit than the null model (χ^2^ = 41.66, p < 0.001). The Hosmer–Lemeshow test confirmed a good model fit (χ^2^ = 119.38, p = 0.7769).

**Table 3:** Binary logistic regression model results on factors associated with postpartum depression levels.^†^

Variable	Categories	OR [95% CI]	P-value
Age (years)	20—29	Reference category	
	≥ 30	0.51 [0.22- 1.18]	0.115
BMI (Kg/m^2^)	Overweight/obese (≥ 25.0)	Reference category	
	Underweight/Normal weight (< 25)	1.19 [0.59- 2.40]	0.632
Education	No college degree	Reference category	
	College degree	**0.39 [0.19- 0.80]**	0.011*
Employment status	Unemployed	Reference category	
	Employed	1.67 [0.78- 3.58]	0.189
General health	Good /fair	Reference category	
	Very good	**0.43 [0.19- 0.98]**	0.044*
	Excellent	**0.21 [0.08- 0.51]**	0.001*
Number of children	One	Reference category	
	More than one	0.40 [0.17- 0.94]	0.034*
Physical activity^£^	Low	Reference category	
	Moderate to High	1.43 [0.73- 2.82]	0.301
Delivery mode^¥^	Cesarean section	Reference category	
	Vaginal delivery	1.52 [0.66- 3.51]	0.326
Breastfeeding duration^**¥**^	≤ 3 months^€^	Reference category	
	> 3 months	**0.46 [0.23- 0.92]**	0.028*

^†^ None-mild PPD level was assigned as the reference level for the analysis.

* Statistically significant at *p*-value < 0.05.

^£^ Participants were categorized into low physical activity (< 600 total MET- minutes/week) vs. moderate-to-high physical activity (≥ 600 total MET- minutes/week).

^¥^ Related to the last delivery.

^€^ Never breastfed or breastfed for 3 months or less.

Results from the regression model showed that -compared to respective reference categories- (OR; 95% CI) earning a college degree (0.39; 0.19- 0.80), having more than one child (0.40; 0.17- 0.94), self-reported very good (0.43; 0.19- 0.98) and excellent health (0.21; 0.08–0.51), and breastfeeding for more than three months (0.46; 0.23–0.92), were significantly associated with reduced odds of moderate-severe PPD. None of the remaining model variables achieved statistical significance (Table 3).

## Discussion

The results of this study suggest that breastfeeding duration is significantly and inversely associated with postpartum depression (PPD) levels among women living in Abu Dhabi, UAE. However, no statistically significant association is found between physical activity and PPD levels. Moreover, education, number of children, self-reported general health, and breastfeeding duration are the only factors significantly associated with PPD levels after conducting a binary logistic regression model. All other factors including the mother’s age, BMI, employment status, physical activity level, and delivery mode, are not significantly associated with PPD level.

### Breastfeeding duration

One-third (33.8%) of the mothers in the current study reported never breastfeeding or breastfeeding for ≤ 3 months; whereas the majority (66.2%) reported breastfeeding for > 3 months (Table 1). As stated previously^14,15,17–19^, the duration of breastfeeding may be a protective factor for PPD, however, according to the global literature, self-report breastfeeding duration varies considerably. In one study^23^, almost half (48%) of new mothers were still breastfeeding 3 months after delivery. Unfortunately, few studies measure duration periods below the 3-month stage; as such, it is more difficult to establish a more detailed duration within this category. This is important given the aforementioned studies suggesting a high proportion of new mothers initiate breastfeeding but this declines rapidly within a few days. Especially if -as we found- a positive association exists between breastfeeding for less than 3 months, and higher self-reported PPD.

### Prevalence of PPD

Approximately one-third (34.8%) of mothers in the present study scored moderate-severe depression, while 65.2% of mothers scored none-mild level of depression, using the EPDS. This concurs with a similar study conducted in Bahrain, also the GCC region, involving 237 Arab women, in which approximately a third (37.1%) of the women self-reported experiencing PPD^37^ In 2015, Haque et al.^38^ reported a prevalence of PPD in new mothers, also in the Middle East, ranging between 10 and 51.8%. As mentioned, classification of mental health, and ill-health, including PPD is highly subjective and therefore problematic; misclassification by health professionals is reported in the literature, moreover, studies suggest that women in this region overestimate reports of depression and anxiety due to greater reliance on symptomatic symptoms; compounding known issues of memory recall in assessing PPD. Since the current study uses retrospective recall of PPD among women with children 3 years and younger, further research accounting for misclassification and recall bias, involving prospective, or considerably shorter retrospective time-scales, is therefore strongly recommended. Despite this, EDPS is globally recognized as a reliable and valid measure of PPD, furthermore, it has been successfully used by retrospective studies, to determine PPD and appears to be stable with time^27,39–41^.

### Factors associated with PPD

Results from Pearson chi-squared test (Table 2) and the binary logistic regression model (Table 3) indicate that number of children, self-reported general health, and breastfeeding duration are significantly and inversely associated with PPD levels. Furthermore, PPD levels are significantly and inversely associated with mother’s age (Pearson chi-squared test; Table 2) and education level (regression model; Table 3).

Number of children is significantly associated with lower PPD levels; significantly higher proportions of mothers giving birth to their first child (50.9%) suffer moderate-severe PPD compared to mothers who have more than one child (28%). Our findings are consistent with other studies that found the number of children may substantially impact self-reported depression scores; this is supported by the literature, which indicates that compared to mothers who have previously had children, first-time mothers are more likely to experience PPD and self-report higher scores^13,40,42^. Additionally, mother’s age is significantly associated with lower PPD levels (Chi-squared p-value = 0.001; Table 2); significantly higher proportions of mothers aged 20–29 years (46.3%) suffer moderate-sever PPD compared to mothers aged 30 years and above (24.8%), which suggests reduction in the proportions reporting moderate-severe PPD with age. This concurs with previous studies, for example, Silverman et al.^43^ concluded higher EPDS score was linked to younger mothers, while the lowest EPDS score was associated with older age i.e. oldest mothers; also Lanzi et al.^44^ concluded that adolescent mothers were more likely to experience and report depression compared with older (adult) mothers. The potential association between self-reported depression, including PPD, with age and number of children is not surprising given what is known about the benefit of knowledge and experience gained naturally through multiple childbirths. Greater consideration when planning public health interventions including targeting messages to new mothers is recommended. Moreover, since we know that the family is valued highly in Emirati culture and social cohesion amongst Arab and Islamic faith populations is reportedly high, mobilizing this to garner support for younger and first-time mothers is important when considering the prevention of PPD in this region.

Self-reported general health is significantly and inversely associated with PPD level. The proportions of mothers who scored moderate-severe PPD were 53.1%, 35%, and 23.3% among mothers who self-reported “Fair/good”, “Very good” and “excellent” general health, respectively. The literature suggests that mother’s general health status is closely associated with PPD^45^, whilst others report that PPD risk increases if mothers have previous medical history or birth complications^46^. Again, whilst this association seems logical, it highlights a potentially protective association, whilst opportunities for targeting mothers with prior medical issues are also recommended.

The present study suggests an inverse association between BF practice and PPD. Half (49.2%) of the mothers who reported never having breastfed or had breastfed their infant for 3-months or less, also scored moderate to severe depression. In contrast, fewer (27.4%) mothers who breastfed for more than 3-months scored moderate to severe depression. This relationship is well supported in the literature. Our findings corroborate previous studies and more importantly add to the limited research within this regional and cultural context. As Hamdan & Tamim reported^40^, mothers who breastfed were less likely to develop PPD, conversely, mothers who didn’t breastfeed were more likely to develop PPD. Another study demonstrated that higher EPDS scores are associated with discontinued breastfeeding 4 months following delivery^14^. Others have reported that the lowest EPDS scores appear for mothers who breastfed non-exclusively and the highest EPDS test score for mothers who didn’t breastfeed at all^17^. In this study, we considered breastfeeding as a whole rather than distinguishing between exclusive and non-exclusive breastfeeding. This approach is consistent with previous research and provides a broad perspective on the association between breastfeeding and PPD. Whilst our findings are supported by similar studies, it is worth noting that the definition of breastfeeding used in the present, and potentially other studies that state using the WHO standards^22^ for defining breastfeeding, did not distinguish between exclusive and non-exclusive breastfeeding. As previously stated, studies have indicated a potential protective effect of breastfeeding against PPD, even when non-exclusive, compared to no breastfeeding^17^. Whilst adopting caution when inferring the strength of this association, we remain optimistic of an association and the benefits of breastfeeding should be researched further. Future studies however should further explore this distinction, using clear criteria on exclusively and duration within a longitudinal study design.

### Physical activity levels & PPD

A considerable body of research indicates an inverse association between Physical Activity (PA) and PPD, however, there was no significant association in the present study. This may be related to the relatively small sample size. Despite evidence of an overall association between PA and PPD, as one comprehensive systematic review^47^ reports, the evidence is far from equivocal, and many studies did not show a significant association between the variables^48–50^. Nonetheless, given the weight of the overall evidence the authors support further exploration of the association between PPD and PA, in particular research into different types and domains of physical activity.

### Strengths and limitations

As mentioned, global estimates of PPD and breastfeeding prevalence vary widely, but especially for this region; therefore, socially and culturally situated studies such as the present are important. The current study is one of a limited number of studies to examine the association between PPD and breastfeeding, undertaken recently in the UAE, and therefore adds to the limited knowledge about this particular population and region. Furthermore, the use of the EPDS, an internationally accepted validated tool for accurately measuring PPD is a major strength; the survey was administered in English and Arabic; the survey prevented double counting by ensuring respondents could access and submit only once. Another strength of our study is the well-defined population, with most participants being Emirati and the vast majority of Middle Eastern origin. This enhances the cultural relevance of our findings and minimizes variability in social and cultural influences on breastfeeding and PPD.

The current study however could have benefited from a larger sample size, and prospective assessment of PPD to reduce potential recall bias. Additionally, the purposive snowball sampling strategy used could increase the potential risk of selection bias. Future studies might also differentiate breastfeeding, exclusive and non-exclusive, with clear criteria on duration while using a longitudinal study design. Lastly, the current study did not assess the broader spectrum of support for breastfeeding—such as that provided by family, community, and healthcare settings. Evidence suggests that comprehensive support can enhance breastfeeding outcomes and reduce PPD risk^51,52^. Future research should explore the impact of these multifaceted support systems on maternal mental health.

In conclusion, this study showed a relatively high prevalence of mothers experiencing moderate to severe PPD. It also demonstrated that longer breastfeeding duration was linked to lower PPD levels. Self-reported general health, number of children, education, and breastfeeding duration were significantly associated with PPD levels, however, the association between PPD and physical activity was not significant. The findings also suggest it may be beneficial to encourage new mothers to breastfeed for a longer period as it could support their psychological health. However, as this study is observational, further research is needed to clarify the direction of this relationship. Future public health messaging should target healthcare professionals, women who are pregnant, and all new mothers with balanced, evidence-based advice on the potential benefits of breastfeeding for mental health.

## Supplementary Information

Supplementary Information.

## Data Availability

The data that support the findings of this study are available from the corresponding author upon reasonable request.
