# Supplementary material for: Breastfeeding is associated with reduction in postpartum depression in the United Arab Emirates: a retrospective cross-sectional study
Source: Sci Rep. 2025 Mar 27;15:10528. doi: 10.1038/s41598-025-94912-3 (PMC11950439; doi:10.1038/s41598-025-94912-3)
Supplement: Supplementary file 1 — Supplementary Information. [file 41598_2025_94912_MOESM1_ESM.docx]

**Supplementary Table S1**. Survey questions with their responses.

| **Sections** | **Questions** | **Answer options** |
| --- | --- | --- |
| 1. **Sociodemographic information** | 1. What is your gender? | - Male. ***Skip to (submit).*** - Female. |
|  | 1. Which Emirate do you live in? | - I don’t live In UAE. ***Skip to (submit).*** - Abu Dhabi. - Dubai. - Sharjah. - Ajman. - Ras Al-Khaimah. - Umm Al Quwain. - Fujairah. |
|  | 1. Do you have any children? | - Yes. - No. ***Skip to (submit).*** |
|  | 1. Is your last child aged three years old or younger? | - Yes. - No. ***Skip to (submit).*** |
|  | 1. What is your nationality? | - Emirati. - Other. |
|  | 1. What is your ethnicity? | - Middle Eastern (e.g. Arab, Persian, Turkish, Kurdish). - Far East Asian (e.g. Chinese, Japanese, Korean). - Southeast Asian (e.g. Indian, Pakistani, Sri Lankan). - African - Caucasian/White. |
|  | 1. What is your age (years)? **(open-ended question)** |  |
|  | 1. What is your height (cm)? (**open-ended question)** |  |
|  | 1. What is your weight in (Kg)? **(open-ended question)** |  |
|  | 1. What is your current marital status? | - Married and/or living with a partner. - Single/never married. - Widowed. - Divorced/Separated. |
|  | 1. What is the highest level of education you have completed? | - Less than high school. - High School. - Associate degree/diploma (2 years) Bachelor's degree. - Higher education (E.g. Master's degree/Ph.D). |
|  | 1. What is your current employment status? | - Unemployed / Housewife. - Full-time employment. - Part-time employment. - Self-employed. - Retired. - Student. |
|  | 1. How would you rate your general health? | - Excellent. - Very good. - Good. - Fair. - Poor. |
| 1. **The Edinburgh Postnatal Depression Scale (EPDS)** | 1. During the first 6 weeks after the birth of your last child, were you able to laugh and see the funny side of things? | - As much as I always (I was able to laugh and see the funny side of things). - Not quite as much. - Definitely not so much. - Not at all (I wasn’t able to laugh and see the funny side of things). |
|  | 1. During the first 6 weeks after the birth of your last child, did you look forward with enjoyment to things? | - As much as I ever did (I looked forward with enjoyment as usual before giving birth). - Rather less than I used to. - Definitely less than I used to. - Hardly at all (I didn’t look forward with enjoyment at all). |
|  | 1. During the first 6 weeks after the birth of your last child, did you blame yourself unnecessarily when things went wrong? | - No, I never blamed myself unnecessarily when things went wrong. - Not very often. - Yes, some of the time. - Yes, most of the time I blamed myself unnecessarily when things went wrong. |
|  | 1. During the first 6 weeks after the birth of your last child, were you anxious or worried for no good reason? | - No, not at all, I wasn’t anxious or worried for no good reason. - Hardly ever. - Yes, sometimes. - Yes, very often I was anxious or worried for no good reason. |
|  | 1. During the first 6 weeks after the birth of your last child, did you feel scared or panicky for no very good reason? | - No, not at all, I didn’t feel scared or panicky for no good reason. - No, not much. - Yes, sometimes. - Yes, quite a lot I felt scared or panicky for no good reason. |
|  | 1. During the first 6 weeks after the birth of your last child, did you feel scared or panicky for no very good reason? | - No, not at all, I didn’t feel scared or panicky for no good reason. - No, not much. - Yes, sometimes. - Yes, quite a lot I felt scared or panicky for no good reason. |
|  | 1. During the first 6 weeks after the birth of your last child, were things getting on top of you? | - No, I was coping as well as ever. - No, most of the time I was coping quite well. - Yes, sometimes I wasn't coping as well as usual. - Yes, most of the time I wasn't able to cope at all. |
|  | 1. During the first 6 weeks after the birth of your last child did you feel so unhappy that it caused you sleeping difficulties? | - No, not at all (I didn’t face sleeping problems because of unhappiness at all). - Not very often. - Yes, sometimes. - Yes, most of the time I had sleeping problems because I was so unhappy. |
|  | 1. During the first 6 weeks after the birth of your last child did you feel sad or miserable? | - No, not at all. - Not very often. - Yes, quite often. - Yes, most of the time. |
|  | 1. During the first 6 weeks after the birth of your last child, did you feel so unhappy that it made you cry? | - No, never (I never felt unhappy that it made me cry). - Only occasionally. - Yes, quite often. - Yes, most of the time I felt so unhappy that it made me cry. |
|  | 1. During the first 6 weeks after the birth of your last child, did you have thoughts of harming yourself? | - Never, I never thought about harming myself. - Hardly ever. - Sometimes. - Yes, quite often I thought about harming myself. |
| 1. **Maternal breastfeeding practice** | 1. How many kids do you have? | - 1 - 2 - 3 - 4 - 5 - 6+ |
|  | 26. What is your last child's gender? | - Male. - Female. |
|  | 27. Delivery type: | - Normal (vaginal). - Cesarean section. |
|  | 28. Are you currently breastfeeding? | - Yes (***Skip to the question 29).*** - No (***Skip to the question 30).*** |
|  | 29. What is your child's age? | - 1-6 days. - 1-3 weeks. - 1-3 months. - 4-6 months. - 6 months to a year. - year to two years. - More than two years old. |
|  | 30. For how long did you breastfeed your last child? | - My baby was never breastfed. ***Skip to the question 31).*** - My baby was breastfed only within the first hours after birth. ***Skip to the question 31).*** - Breastfeeding lasted only 1-6 days. (***Skip to the question 31).*** - Breastfeeding lasted 1-3 weeks. (***Skip to the question 31).*** - Breastfeeding lasted 1-3 months. (***Skip to the question 31).*** - Breastfeeding lasts from 4-6 months. (***Skip to the question 31).*** - Breastfeeding lasts from 6 months to a year. (***Skip to the question 32).*** - Breastfeeding lasted less than two years. (***Skip to the question 32).*** - Breastfeeding continued for two years. (***Skip to the question 32).*** |
|  | 31. Which of the following explains why you decided to stop breastfeeding your child? **(choose all that apply question)** | - My baby had trouble sucking or latching on. - My baby was born prematurely (when a baby is born too early). - Doctor recommendation. - My baby became sick and could not breastfeed. - My baby began to bite. - My baby lost interest in breastfeeding or began to wean himself/herself. - Baby was not gaining enough weight. - I didn’t have enough milk. - Nipples were sore, cracked, or bleeding. - Breasts were overfull or engorged. - Breasts were infected or abscessed. - Breasts leaked too much. - Breastfeeding was too painful. - Breastfeeding was too tiring. - I became pregnant or wanted to become pregnant again. - I did not like breastfeeding. - Breastfeeding was too inconvenient. - I wanted to be able to leave my baby for several hours at a time. - I wanted to go on a weight-loss diet. - I wanted to go back to my usual diet. - I had too many household duties. - I could not or did not want to pump or breastfeed at work. - I was not present to feed my baby for reasons other than work. |
| 1. **Physical activity** | **Vigorous Activities**  32. During the first 3 - 6 weeks after your last delivery, how many days a week did you do vigorous physical activities like *heavy lifting, digging, aerobics, or fast bicycling?* | - None. (***Skip to question 34*** ) - 1 - 2 - 3 - 4 - 5 - 6 - 7 |
|  | 33. During the first 3-6 weeks after your last delivery, on average how much time did you spend doing vigorous physical activities on one of those days? | - 15 minutes - 30 minutes - 45 minutes - 1hour - 2hours - 3 hours - 4 hours - 5 hours - 6+ hours |
|  | **Moderate Activities:**  34. During the first 3-6 weeks after your last delivery, how many days a week did you do Moderate physical activities like *carrying light loads, bicycling at a regular pace running, or doubles tennis?* | - None. **(Skip to question 36)** - 1 - 2 - 3 - 4 - 5 - 6 - 7 |
|  | 35. During the first 3-6 weeks after your last delivery, on average how much time did you spend doing moderate physical activities on one of those days? | - 15 minutes - 30 minutes - 45 minutes - 1hour - 2hours - 3 hours - 4 hours - 5 hours - 6+ hours |
|  | **Walking:**  36. During the first 3-6 weeks after your last delivery, on average, how much time did you spend walking every day? | - 15 minutes - 30 minutes - 45 minutes - 1hour - 2hours - 3 hours - 4 hours - 5 hours - 6+ hours |
|  | **Sitting:**  37. During the first 3-6 weeks after your last delivery, on average, how much time did you spend walking every day? | - 15 minutes - 30 minutes - 45 minutes - 1hour - 2hours - 3 hours - 4 hours - 5 hours - 6+ hours |
